# Functional Implications of Ubiquitous Semicircular Canal Non-Orthogonality in Mammals

**DOI:** 10.1371/journal.pone.0079585

**Published:** 2013-11-19

**Authors:** Jeri C. Berlin, E. Christopher Kirk, Timothy B. Rowe

**Affiliations:** 1 Vertebrate Paleontology Laboratory, University of Texas at Austin, Austin, Texas, United States of America; 2 Department of Anthropology, University of Texas at Austin, Austin, Texas, United States of America; 3 Jackson School of Geosciences, University of Texas at Austin, Austin, Texas, United States of America; Raymond M. Alf Museum of Paleontology, United States of America

## Abstract

The ‘canonical model’ of semicircular canal orientation in mammals assumes that 1) the three ipsilateral canals of an inner ear exist in orthogonal planes (i.e., orthogonality), 2) corresponding left and right canal pairs have equivalent angles (i.e., angle symmetry), and 3) contralateral synergistic canals occupy parallel planes (i.e., coplanarity). However, descriptions of vestibular anatomy that quantify semicircular canal orientation in single species often diverge substantially from this model. Data for primates further suggest that semicircular canal orthogonality varies predictably with the angular head velocities encountered in locomotion. These observations raise the possibility that orthogonality, symmetry, and coplanarity are misleading descriptors of semicircular canal orientation in mammals, and that deviations from these norms could have significant functional consequences. Here we critically assess the canonical model of semicircular canal orientation using high-resolution X-ray computed tomography scans of 39 mammal species. We find that substantial deviations from orthogonality, angle symmetry, and coplanarity are the rule for the mammals in our comparative sample. Furthermore, the degree to which the semicircular canals of a given species deviate from orthogonality is negatively correlated with estimated vestibular sensitivity. We conclude that the available comparative morphometric data do not support the canonical model and that its overemphasis as a heuristic generalization obscures a large amount of functionally relevant variation in semicircular canal orientation between species.

## Introduction

Detection of angular head accelerations is mediated by the semicircular canals of the inner ear. Each semicircular canal consists of a toroidal bony passage within the petrous portion of the temporal bone and contains an endolymph-filled duct. When the head rotates, inertial drag of endolymph within the duct acts upon sensory hair cells that modulate the firing rates of primary vestibular afferent neurons. Firing rates are either increased or decreased depending on the direction of head rotation [1]. The excitatory and inhibitory signals from all six semicircular canals are combined in the brain to generate reflexive movements that help to stabilize the eyes and head when the body is in motion [2].

**Figure 1:**
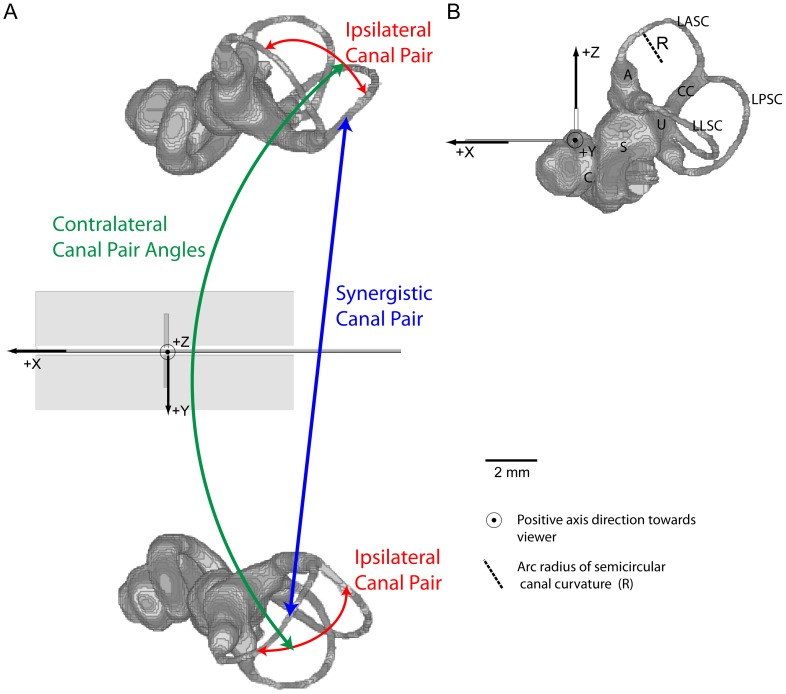
Bony inner ear endocast of *Petauroides volans* (AMNH 150055) showing embedded head-centered reference planes and SCC canal pair types. A, dorsal view showing X, Y, and Z axes. Center point occurs at intersection of all three planes. Axial plane = YZ reference plane passing through the interaural line; frontal plane = XY reference plane defined by Reid's Plane (perpendicular to viewer); sagittal plane = XZ reference plane passing through central features. B, Left lateral view with sagittal plane perpendicular to viewer. Abbreviations: A, ampulla; C, cochlea; CC, common crus; LASC, left anterior semicircular canal; LLSC, left lateral semicircular canal; LPSC, left posterior semicircular canal; R, arc radius of curvature of the left anterior semicircular canal; S, saccule; U, utricle.

In contrast with more readily accessible peripheral sense organs like the eye, the fact that the inner ear is encased within dense bone has hampered the comparative study of semicircular canal anatomy. This limitation, combined with the assumption that the three canals in each inner ear evolved to optimally detect rotations in each of the three orthogonal spatial dimensions, led researchers to rely on a series of simplifying assumptions about semicircular canal anatomy that are seldom critically examined. According to this “canonical model” of semicircular canal morphology, the plane of each canal is orthogonal to the planes of the other two ipsilateral canals so that all three canals in a single inner ear intersect at 90° angles [3]–[9]. Furthermore, contralateral semicircular canals are assumed to be essentially identical in dimension and orientation [4], [10]. As a result, corresponding left and right canal pairs are expected to have equivalent angles and contralateral synergistic canals are expected to occupy parallel planes (Figure 1). These three basic components of the model, including orthogonality, angle symmetry, and coplanarity, are stated explicitly or implicitly in nearly every textbook or academic review covering the vestibular system [11]–[16]. Nonetheless, some empirical studies that measured semicircular canal orientation in a limited range of species reported results that are considerably divergent from the canonical model [e.g. 17], [18]–[23]. Humans, for example, are reported to have ipsilateral canal pairs that differ by as much as 22° from orthogonality [17].

The comparative morphology of semicircular canals is important because canal orientation ostensibly influences vestibular function [24]–[26]. However, most comparative analyses have examined the relationship between semicircular canal radius of curvature and locomotor agility [27]–[31]. Although radius of curvature is a major determinant of the sensitivity in each canal, the orientations of all six canals also help determine the relative sensitivity of the vestibular system to angular accelerations in three dimensions [15], [24]–[26], [32].

As a result, some authors have incorporated canal orientation in their calculations of vestibular sensitivity to angular accelerations [25], [33], [34]. To date, the largest comparative analysis of three-dimensional vestibular sensitivity focused on semicircular canal morphology and locomotor kinetics in 11 species of strepsirrhine primates [35]. This study found substantial variation between species in the homogeneity of three dimensional sensitivity maps, and demonstrated that strepsirrhines with more orthogonal canals tend to encounter higher angular head velocities during locomotion. More broadly, this analysis also provided evidence that deviations from canal orthogonality have important consequences for vestibular function.

In contrast to early studies of vestibular anatomy that relied on gross dissection or histology (11, 12, 19, 22, 28–30), computed tomography is now the standard for studies of semicircular canal morphology because it is nondestructive, quantitative, and can provide excellent resolution of internal cranial spaces [36], [37]. We used high-resolution x-ray computed tomography scans of bilateral inner ear labyrinths to quantify semicircular canal size and orientation in 39 extant species from 11 mammalian orders (Table 1). For each taxon in our sample, these data were used to quantify mean deviations of ipsilateral semicircular canal pairs from orthogonality (90_var_), the degree to which corresponding contralateral canal pair angles differ (Angle Symmetry_dev_), and the degree to which synergistic canal pairs deviate from coplanarity (Coplanarity_dev_). The term 90_var_ was introduced by Malinzak et al. as the sum of the absolute value of the difference between each of three unilateral ipsilateral canal pair angle and 90° [35]. Here we calculate 90_var_ by summing the absolute value of the difference between all six ipsilateral semicircular canal pair angles and 90°, and taking the mean (see below).

**Table 1:** Taxa, museum specimen number, High Resolution X-ray Computed Tomography image slices used for skull images, spacing between image slices in image stack, Field of Reconstruction, and image slice pixel size.

Taxon	Common Name	Museum Specimen Number	Number of slices	Interslice Spacing (mm)	Field of Recon-struction (mm)	File size (pixels)
*Acrobates pygmaeus*	Pygmy Gliding Possum	AMNH 155057	406	0.03	28.0	1024×1024
*Allactaga major*	Five-toed Jerboa	AMNH 178795	1170	0.04	37.0	1024×1024
*Anomalurus beecrofti*	Scaly-tailed Flying Squirrel	AMNH 50483	1270	0.04	39.0	1024×1024
*Caluromys* sp.	Woolly Opossum	AMNH 95526	746	0.08	38.0	1024×1024
*Cavia porcellus*	Guinea Pig	TMM M-7283	1524	0.04	42.0	1024×1024
*Cercartetus caudatus*	Dormouse Possum	AMNH 155090	705	0.04	18.0	1024×1024
*Chinchilla laniger*	Chinchilla	Hullar	1887	0.04	40	1024×1024
*Chironectes minimus*	Water Opossum	AMNH 129701	1522	0.04	33.0	1024×1024
*Chrysochloris* sp.	Golden Mole	AMNH 82372	513	0.05	31.0	1024×1024
*Crocuta crocuta*	Hyena	UCMVZ 184551	528	0.50	166.0	512×512
*Dactylopsila trivirgata*	Striped Possum	AMNH 104040	1301	0.05	45.0	1024×1024
*Dolichotis patagonum*	Patagonian Hare	AMNH 80078	1705	0.07	56.2	1024×1024
*Dromiciops gliroides*	Monito del Monte	FMNH 127463	711	0.04	16.6	1024×1024
*Enhydra lutris*	Sea Otter	SO 2853-97	645	0.22	106.0	1024×1024
*Felis catus*	Domestic Cat	TMM M-628	606	0.15	70.0	1024×1024
*Glaucomys volans*	Eastern Flying Squirrel	TMM M-6332	474	0.08	22.9	522×522
*Hemibelideus lemuroides*	Brush-tipped Ring-tailed Possum	AMNH 154375	1207	0.05	40.0	1024×1024
*Heterocephalus glaber*	Naked Mole Rat	AMNH 113974	1050	0.02	21.0	1024×1024
*Lepus californicus*	Hare	TMM M-7500	660	0.14	67.0	1024×1024
*Meriones unguiculatus*	Gerbil	TMM M-05306	1394	0.02	23.0	1024×1024
*Monodelphis domestica*	Short-tailed Opossum	TMM M-9039	885	0.14	21.0	1024×1024
*Mus musculus*	House Mouse	TMM M-3196	737	0.03	13.5	1024×1024
*Notoryctes typhlops*	Marsupial Mole	AMNH 202107	705	0.04	18.0	1024×1024
*Ornithorhynchus anatinus*	Duck-billed Platypus	TMM M-5899	1998	0.05	43.0	1024×1024
*Pedetes capensis*	Springhare	AMNH 42016	1145	0.07	67.0	1024×1024
*Petauroides volans*	Greater Gliding Possum	AMNH 150055	1251	0.05	48.0	1024×1024
*Petaurus breviceps*	Sugar Glider	TMM M-8226	555	0.07	33.0	1024×1024
*Petropseudes dahli*	Rock Possum	AMNH 183391	1424	0.05	46.0	1024×1024
*Potorous tridactylus*	Long-nosed Potoroo	AMNH 65337	915	0.01	48.0	1024×1024
*Pseudocheirus peregrinus*	Common Ring-tailed Possum	TMM M-847	795	0.09	43.0	1024×1024
*Pseudochirops cupreus*	Coppery Ring-tailed Possum	AMNH 151829	1289	0.05	49.5	1024×1024
*Pseudochirulus forbesi*	New Guinean Ring-tailed Possum	AMNH 104136	1339	0.03	33.0	1024×1024
*Rattus norvegicus*	Brown Rat	TMM M-2272	1571	0.03	28.0	1024×1024
*Saimiri sciureus*	Squirrel Monkey	NSm7	310	0.07	64.0	1024×1024
*Sciurus niger*	Fox Squirrel	UMMZ 123729	450	0.16	44.4	512×512
*Talpa europaea*	Old World Mole	UCLGMZ 5437	585	0.06	18.5	1024×1024
*Tarsipes rostratus*	Honey Possum	AMNH 119717	921	0.03	13.0	1024×1024
*Vulpes vulpes*	Red Fox	UCLA 13112	825	0.17	80.0	1024×1024
*Wallabia bicolor*	Swamp Wallaby	TMM M-4169	885	0.16	74.5	1024×1024

Museum Abbreviations: **AMNH**, American Museum of Natural History, New York; **FMNH**, Field Museum of Natural History, Chicago; **TMM**, Texas Natural Science Centers, Vertebrate Paleontology, Austin; **UCL GMZ**, University College, London Grant Museum of Zoology; **SO**, University of California Los Angeles Museum; **UCLA**, University of California Los Angeles; **UCMVZ**, University of California Museum of Vertebrate Zoology, Berkeley; **UMMZ**, University of Michigan Museum of Zoology, Ann Arbor.

We also used bilateral measurements of the size and orientation of all six semicircular canals to estimate the maximum (Sensitivity_max_) and average (Sensitivity_ave_) sensitivity of the vestibular system to angular accelerations in three dimensions. These data for a large and taxonomically diverse sample allowed us to examine the degree to which orthogonality, symmetry, and coplanarity are characteristic of mammalian semicircular canals and to determine whether deviations from these norms are correlated with interspecific differences in estimated vestibular sensitivity.

## Materials and Methods

The crania of 39 extant mammals, each representing a different genus, were scanned at the University of Texas High-Resolution X-ray Computed Tomography Facility (Austin, Texas). This facility maintains an archive of all scans used in this analysis. Taxon, museum specimen number, and scan parameters for each cranium used in this study are listed in Table 1. With the exception of *Chinchilla*, for which a preexisting scan was made available by Dr. Timothy Hullar, all cranial specimens used in this analysis were scanned with the permission of the museums listed in Table 1. All crania were scanned bilaterally, ensuring that both the right and left semicircular canals were scanned in situ. This bilateral scanning protocol allowed measurements of contralateral canal pairs, a parameter that is rarely measured. Bilateral scanning also permitted the calculation of the vestibular sensitivity of each specimen to head rotations in three dimensions. The resulting image stacks were imported into VGStudioMax© (Versions 1.2 and 2.0; Volume Graphics GmbH, 2004 and 2007) for 3D imaging and analysis.

For the present study, canal angle comparisons required stable head-centered reference planes, especially for angle comparison of contralateral canals. Three reference planes were determined and segmented into the 3D digital images before any other analysis was undertaken. The terminology follows that of vestibular researchers [e.g.], [ 22], [24,26]. Approximately eight small reference segments along the median sutures of the skull images (e.g., nasals, nasion, bregma, and medial palatine sutures) were aligned in a best-fit plane to define the vertical sagittal (XZ) plane. Numerous previous authors assumed that the LSC represents the horizontal plane of a live animal's head orientation, thus the alternative designation of the canal as the horizontal semicircular canal [e.g.], [ 26], [38,39], but that also assumed that both the left and right lateral canals lie within the same horizontal plane. Because this assumption is not correct (see below), we used bilateral measurements of Reid's line (the line extending from the lower edge of the orbit to the center of the aperture of the external auditory canal [40] to define the horizontal/frontal plane (XY). The axial (YZ) plane contained the line connecting the two external auditory meatuses (interaural line) perpendicular to the frontal and sagittal reference planes. The positive X axis of the resultant head-centered reference system passed through the rostrum, the positive Y axis passed through the left meatus, and the positive Z axis passed dorsally through the skull. Such a coordinate system was fitted successfully to all specimens except a *Thylacinus*, which was discarded for phylogenetic purposes.

Images were thresholded in VGStudioMax based on the density of the petrosal using the VGStudio density averaging tool. The selected region of the bony labyrinth was subsequently outlined for each CT slice image in VGStudioMax and added together to produce segments representing endocasts of both bony labyrinths in each specimen. A resulting file of the reference planes and bony labyrinth endocasts for *Petauroides volans* is shown in Figure 1. For our determination of canal orientations with regard to the reference planes in VGStudioMax, we used a measurement tool to select an array of points (∼60–100 per canal) representing the lumen centers of a canal from the end of the ampulla, around the canal and including the common crus. A circle circumference for each canal was calculated by a linear regression best-fit of the selected lumen points. The radius to the **s**emicircular canal circumference (R, in mm) was recorded for use in sensitivity calculations. The fitpoints were imported into a VGStudioMax best-fit calculation to obtain the plane containing that semicircular canal [19], defined by coordinates of the unit normal axis perpendicular to that plane. A plane's normal line has no polarity, but each semicircular canal can be rotated in a direction that provides an increase in afferent neuron firing rate (excitatory direction) or it may be rotated in the opposite direction to produce a decrease in afferent neuron firing rate (inhibitory direction). To express this additional information, the normal line, serving as an axis of rotation, was polarized to give a vector (V) showing excitatory sensitivity direction according to the right-hand rule as described by Ezure and Graf [19] and utilized by Calabrese and Hullar [22]. Mathematical calculation of angles between all canals was performed in VGStudioMax, with corrections to ensure all angles are internal (in lateral direction of skull) [see 19,41]. Naming convention of the angles closely follows that of Spoor and Zonneveld [42]. For example, LASC∡LLSC refers to the angle between the left anterior semicircular canal and the left lateral semicircular canal. All angles measured for each species are listed in Table 2, and summary data for ipsilateral canal angles and synergistic contralateral canals are provided in Table 3. The summary angular data in Table 3 includes both arithmetic means with standard deviations, as well as mean directions with circular standard deviations calculated by treating our data as vectors [43]. The arithmetic mean and mean direction for these data demonstrated negligible differences (i.e., ≦0.02°), while the circular standard deviation is less than the arithmetic standard deviation (Table 3). In all analyses, our angular measurements were quantified as the absolute value of the deviation from an expected value (either 90° or 180°; see below). Although these measurements are expressed in degrees, the data used in all analyses are scalar and do not require the use of circular statistics.

**Table 2:** Summary of semicircular canal angle relationships.

Taxon	LASC ∡LLSC IPS	LASC ∡LPSC IPS	LLSC ∡LPSC IPS	RASC ∡RLSC IPS	RASC ∡RPSC IPS	RLSC ∡RPSC IPS	LASC ∡RPSC SYN	LPSC ∡RASC SYN	LLSC ∡RLSC SYN
*Acrobates*	103.86	90.54	81.01	98.35	89.17	86.43	7.63	9.86	8.95
*Allactaga*	72.26	82.03	86.86	71.09	83.50	84.69	19.58	19.55	10.55
*Anomalurus*	86.65	104.52	97.34	88.10	102.05	97.77	4.39	7.52	1.44
*Caluromys*	83.19	96.48	89.63	77.14	97.75	87.54	10.36	10.03	26.72
*Cavia*	82.29	86.47	85.12	88.99	87.10	84.44	7.11	6.69	1.19
*Cercartetus*	84.15	91.73	87.35	81.53	87.41	84.80	9.97	7.99	25.90
*Chinchilla*	86.19	84.87	83.58	74.51	87.09	81.58			
*Chironectes*	82.09	104.33	91.91	77.84	93.55	93.34	19.10	11.69	20.25
*Chrysochloris*	58.82	86.41	103.87	71.65	84.12	91.58	15.48	10.06	14.71
*Crocuta*	80.54	95.71	99.05	81.39	94.32	90.01	3.59	8.48	13.20
*Dactylopsila*	82.82	89.83	94.77	88.04	95.08	96.03	9.25	2.69	7.16
*Dolichotis*	88.13	89.25	88.36	74.61	75.16	89.79	14.26	1.58	17.54
*Dromiciops*	92.87	89.71	92.39	88.75	101.19	94.69	19.17	14.68	15.72
*Enhydra*	89.35	98.42	91.64	91.16	102.79	83.18	3.91	0.51	8.55
*Felis*	87.27	83.62	81.94	83.79	84.62	81.65	12.57	11.53	10.75
*Glaucomys*	86.71	90.28	94.48	82.19	87.17	89.96	5.66	12.71	18.26
*Hemibelideus*	83.86	105.74	91.00	78.14	102.18	85.44	10.91	9.13	3.27
*Heterocephalus*	79.61	96.08	96.45	77.65	90.61	97.21	10.84	10.96	20.34
*Lepus*	87.92	93.87	87.13	87.87	95.84	87.45	4.60	5.19	0.61
*Meriones*	88.92	87.66	82.46	84.03	89.03	86.05	1.66	2.55	4.49
*Monodelphis*	83.90	95.99	91.06	81.31	99.56	89.32	15.05	17.04	24.19
*Mus*	85.82	78.08	85.21	85.84	84.89	85.26	6.09	10.45	9.91
*Notoryctes*	78.61	121.21	93.47	68.23	107.69	82.78	6.74	9.29	17.44
*Ornithorhynchus*	82.12	80.10	82.98	75.70	82.23	88.63	4.56	6.70	21.67
*Pedetes*	94.52	93.63	89.23	91.36	93.12	89.25	6.64	2.63	3.00
*Petauroides*	82.95	98.23	85.29	79.67	93.42	86.31	11.04	15.03	22.29
*Petaurus*	80.42	89.18	98.58	86.01	91.33	102.68	2.00	4.85	3.21
*Petropseudes*	76.77	89.07	93.20	89.48	89.09	90.86	15.74	14.14	3.61
*Potorous*	91.64	88.35	88.41	94.76	87.65	88.32	5.74	4.63	5.01
*Pseudocheirus*	84.86	93.67	92.90	84.10	98.52	90.41	19.13	24.15	6.42
*Pseudochirops*	93.28	83.83	104.81	87.93	87.37	85.92	11.87	15.29	9.53
*Pseudochirulus*	92.18	104.50	98.10	89.71	101.19	103.14	9.93	2.32	7.64
*Rattus*	88.64	87.63	79.72	84.10	87.33	81.97	11.65	9.96	8.22
*Saimiri*	85.97	79.77	89.55	87.26	76.24	88.10	7.97	12.29	5.52
*Sciurus*	79.26	86.88	90.60	84.04	82.88	91.93	5.54	1.71	6.10
*Talpa*	76.67	105.11	89.71	78.48	91.73	100.71	7.09	7.47	8.21
*Tarsipes*	97.36	104.25	92.06	101.85	98.42	90.51	10.04	4.21	10.24
*Vulpes*	87.46	83.76	97.34	88.49	91.52	102.77	8.02	12.97	5.14
*Wallabia*	85.39	87.79	92.37	89.71	93.25	91.73	11.82	18.59	19.54
Mean	85.01	92.27	90.79	83.97	91.47	89.85	9.65	9.40	11.22
Standard Deviation	7.40	8.91	5.97	7.32	7.38	5.89	4.90	5.52	7.51

Includes angles between all three pairs of ipsilateral for left and right sides, angles between left and right contralateral angle pairs, and angles between three synergistic canal pairs, all in degrees. IPS: ipsilateral canal pair angle, SYN: synergistic canal pair angle.

**Table 3:** Summary data for the 39 mammalian species in the comparative sample.

	ASC ∡LSC IPS	ASC ∡PSC IPS	LSC ∡PSC IPS	ASC ∡PSC SYN	LSC ∡LSC SYN
Arithmetic Mean	84.49	91.87	90.32	9.52	11.22
Arithmetic Standard Deviation	7.33	8.14	5.91	5.19	7.51
Mean Direction	84.50	91.85	90.32	9.52	11.21
Circular Standard Deviation	4.80	5.32	3.87	3.40	4.88

IPS: ipsilateral canal pair angle, SYN: synergistic canal pair angle. All angles in degrees.

### Orthogonality, Symmetry, and Coplanarity Calculations

We quantified semicircular canal orientation by comparing the deviations of canal pair angles from the expected normative values. Deviation from orthogonality (90_var_) [35] was calculated by taking the absolute value of the difference between each canal pair angle and 90°, adding those difference for all six ipsilateral canal pairs, and dividing by six. Deviation from side to side semicircular canal angle symmetry (Angle Symmetry_dev_) was calculated as the absolute value of the difference between the left canal pair angle and the corresponding right canal pair angle. To quantify deviation from coplanarity (Coplanarity_dev_) we first subtracted the angle between each synergistic contralateral canal pair from 180°. We then summed the absolute value of this difference for each of the three synergistic canal pairs and divided by 3. Values for these variables are given in Table 4.

**Table 4:** Deviations from orthogonality (90_var_), side-to-side angle symmetry (Angle Symmetry_dev_), and synergistic canal coplanarity (Coplanarity_dev_).

Taxon	90_var_	Angle Symmetry_dev_	Coplanarity_dev_	S_max_	S_ave_	OS_max_	OS_ave_
*Acrobates*	6.02	4.10	8.83	0.42	0.34	0.39	0.34
*Allactaga*	9.93	1.60	16.56	1.35	1.05	1.22	1.05
*Anomalurus*	7.82	1.45	4.44	1.23	0.99	1.14	0.99
*Caluromys*	6.12	3.13	15.70	0.68	0.56	0.65	0.56
*Cavia*	4.27	2.67	5.00	1.17	0.97	1.12	0.97
*Cercartetus*	4.42	3.16	14.32	0.49	0.34	0.40	0.34
*Chinchilla*	7.03	5.30		1.39	1.10	1.27	1.1
*Chironectes*	7.20	5.49	17.01	0.71	0.59	0.68	0.59
*Chrysochloris*	12.41	9.13	13.41	0.38	0.27	0.32	0.27
*Crocuta*	6.20	3.76	8.41	2.17	1.78	2.07	1.78
*Dactylopsila*	4.20	3.91	6.40	0.87	0.72	0.84	0.72
*Dolichotis*	5.78	9.68	11.13	1.62	1.30	1.50	1.3
*Dromiciops*	3.78	5.97	16.53	0.38	0.32	0.37	0.32
*Enhydra*	5.25	4.88	4.36	1.62	1.30	1.50	1.3
*Felis*	6.19	1.59	11.62	1.42	1.07	1.24	1.07
*Glaucomys*	3.12	4.05	12.21	0.88	0.74	0.85	0.74
*Hemibelideus*	8.58	4.95	7.79	1.52	1.17	1.37	1.17
*Heterocephalus*	7.18	2.73	14.04	0.41	0.33	0.39	0.33
*Lepus*	3.22	0.78	3.48	1.35	1.13	1.31	1.13
*Meriones*	3.64	3.28	2.90	0.83	0.68	0.78	0.68
*Monodelphis*	5.35	2.64	18.76	0.46	0.37	0.44	0.37
*Mus*	5.82	2.29	8.82	0.26	0.21	0.25	0.21
*Notoryctes*	15.45	11.55	11.14	0.38	0.27	0.32	0.27
*Ornithorhynchus*	8.04	4.73	10.98	1.12	0.90	1.05	0.9
*Pedetes*	2.36	1.23	4.05	1.58	1.32	1.53	1.32
*Petauroides*	6.24	3.04	16.12	1.11	0.92	1.07	0.92
*Petaurus*	6.16	3.95	3.39	0.73	0.58	0.67	0.58
*Petropseudes*	3.27	5.02	11.16	1.16	0.93	1.08	0.93
*Potorous*	2.28	1.31	5.11	1.20	0.99	1.15	0.99
*Pseudocheirus*	4.42	2.70	16.57	1.16	0.95	1.11	0.95
*Pseudochirops*	5.51	9.26	12.23	1.11	0.92	1.07	0.92
*Pseudochirulus*	8.24	3.61	6.63	1.00	0.79	0.92	0.79
*Rattus*	5.10	2.36	9.94	0.75	0.63	0.73	0.63
*Saimiri*	5.52	2.09	10.13	1.32	1.05	1.21	1.05
*Sciurus*	4.91	3.37	4.47	1.65	1.32	1.53	1.32
*Talpa*	8.78	8.73	7.57	0.59	0.48	0.56	0.48
*Tarsipes*	7.41	8.86	8.15	0.26	0.20	0.23	0.2
*Vulpes*	5.32	4.74	8.72	1.62	1.30	1.51	1.3
*Wallabia*	2.41	3.47	16.66	1.81	1.55	1.81	1.55

Sensitivities calculated from canal dimensions [32] and angles from Table 2. Hypothetical ‘Orthogonal Sensitivity’ calculated by forcing all canal angles to be orthogonal and symmetrical, but keeping all R dimensions as originally measured.

### Sensitivity Calculations

**Figure 2:**
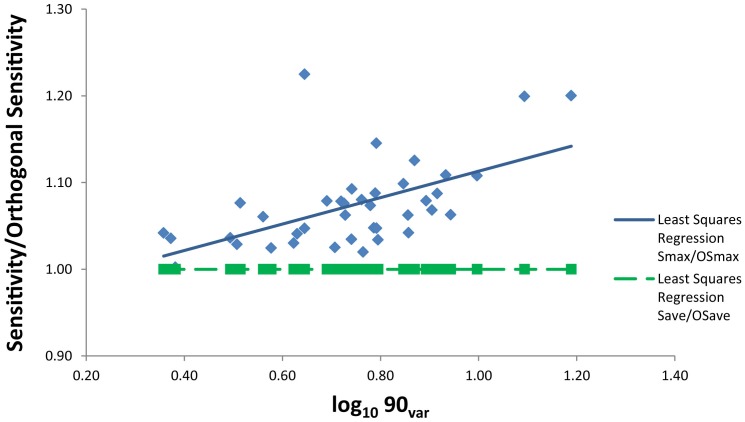
Effect of constraining semicircular canals to strict orthogonality. This plot shows the relationship between 90_var_ (x-axis) and two ratios (y-axis): (1) Maximum observed sensitivity (S_max_) : Maximum sensitivity with orthogonality constrained (OS_max_) (blue diamonds), and (2) Average observed sensitivity (S_ave_) : Average sensitivity with orthogonality constrained (OS_ave_) (green squares). Data from Table 4.

A rotation of the head in the plane of a given semicircular canal (i.e. around V) increases the firing rate of the vestibular nerve cells in that canal above a resting rate (in spikes · sec^−1^), or decreases the firing rate (axis opposite to V). The rate of nerve cell firing change is proportional to R and the speed of rotation (in degrees · sec^−1^), and is referred to as the sensitivity of the canal to rotation (in spikes · sec^−1^/degrees · sec^−1^) [16], [24], [25], [32]. A head rotation around an axis with orientation X changes the sensitivity of the canal nerve responses in proportion to the cosine of the angle between X and V. The responses of all six individual semicircular canals to rotation along X can be determined with R and V for each canal, and the orientation of X. Therefore, for a head rotation along any head-centered axis X, the sensitivities of all six canals can be summed to provide a global sensitivity to the rotation (S, in spikes · sec^−1^/degrees · sec^−1^). By calculating such global sensitivities for a high number of rotational axes, an axis of rotation resulting in the maximum summed sensitivity of all six canals can be determined (Sensitivity_max_). The average sensitivity for a high number of rotational axes can also be calculated (Sensitivity_ave_). The Sensitivity_max_ and Sensitivity_ave_ values calculated for specimens used in this study are listed in Table 4. Both sensitivity values were calculated using Bubbles.mat [35] software, which is described in detail by Yang and Hullar [25], Rodgers [32], and Malinzak et al. [32]. Bubbles.mat uses both the orientation and radius of curvature of the six semicircular canals to calculate estimated sensitivity of the vestibular system to angular accelerations in three dimensions. According to the Bubbles.mat results, the calculated Sensitivity_max_ is a function of both canal orientations and canal radii but Sensitivity_ave_ is entirely determined by canal radii. These effects are illustrated in Figure 2, which shows the relationship between 90_var_ and the ratio of our observed sensitivities to sensitivity calculated with canals constrained to strict orthogonality (OSensitivity_max_ and OSensitivity_min_) through setting all ipsilateral canal angles as 90° bilaterally, and setting ASC and PSC 45° away from the sagittal plane for Bubbles.mat calculations. In Figure 2, the ratio of Sensitivity_ave_ to OSensitivity_ave_ is always 1.0 across a range of 90_var_ values which indicates that Sensitivity_ave_ is solely a function of R. By comparison, the ratio of Sensitivity_max_ to OSensitivity_max_ is always greater than 1.0 and tends to increase with increases in 90_var_ (Figure 2). This ratio of Sensitivity_max_ ∶ OSensitivity_max_ reflects the fact that artificially constraining strict orthogonality leads to a decrease in the maximum estimated sensitivity of the vestibular system according to the Bubbles.mat calculations. The implications of these effects for our analyses of canal orientation an estimated sensitivity are discussed below.

### Statistical Analysis

All statistical tests were performed in R using the ape and nlme packages [44]–[46]. A normal distribution for all continuous data was confirmed using Shapiro-Wilk tests for normality. Raw data for 90_var_ and Angle Symmetry_dev_ deviated significantly from normality, so these data were log10 transformed to satisfy the assumptions of parametric statistical tests. A normal distribution for both 90_var_ and Angle Symmetry_dev_ following log10 transformation was confirmed with a Shapiro-Wilk test. Data for Coplanarity_dev_, Sensitivity_max_, and Sensitivity_ave_ did not deviate significantly from normality and were therefore included in analyses without transformation.

Two types of statistical tests were used assess the relationship between the morphology and estimated sensitivity of the semicircular canals. First, Pearson product-moment correlations were calculated for our three measures of canal morphology (90_var_, Angle Symmetry_dev_, and Coplanarity_dev_) and our two estimates of sensitivity to angular accelerations in three dimensions (Sensitivity_max_ and Sensitivity_ave_). (Table 5). Because we expect deviations from orthogonality (i.e., greater 90_var_ values), deviations from equality in corresponding contralateral angle pairs (i.e., greater Angle Symmetry_dev_ values), and deviations from coplanarity in synergistic canal pairs (i.e., greater Coplanarity_dev_ values) to be negatively correlated with vestibular sensitivity, all correlations were one-tailed. Second, phylogenetic generalized least-squares regression (PGLS) [47] was used to examine the relationships between canal morphology and estimated sensitivity while controlling for phylogenetic relationships. Tree topology and branch lengths for the included taxa follow Bininda-Emonds et al. [48], [49]. The strength of the phylogenetic signal (i.e., the degree to which data approximate a Brownian-motion model of evolution) in each PGLS analysis was quantified using Pagel's lambda (λ) [50].

**Table 5:** Results of Statistical Tests.

	90_var_	Angle Symmetry_dev_	Coplanarity_dev_
S_ave_	Pearson: P = 0.019*; r = −0.335 PGLS: P = 0.029*; λ = 0.648	Pearson: P = 0.047*; r = −0.272 PGLS: P = 0.236; λ = 0.636	Pearson: P = 0.041*; r = −0.286 PGLS: P = 0.363; λ = 0.762
S_max_	Pearson: P = 0.036*; r = −0.292 PGLS: P = **0.060**; λ = 0.682	Pearson: P = **0.054**; r = −0.262 PGLS: P = 0.273; λ = 0.684	Pearson: P = 0.046*; r = −0.276 PGLS: P = 0.372; λ = 0.791

P-values for significant results and non-significant trends shown in bold; Results significant at P<0.05 marked with an asterisk. “Pearson” = one-tailed Pearson product-moment correlation, “PGLS” = phylogenetic generalized least-squares regression.

## Results

### Deviations from orthogonality, angle symmetry, and coplanarity

**Figure 3:**
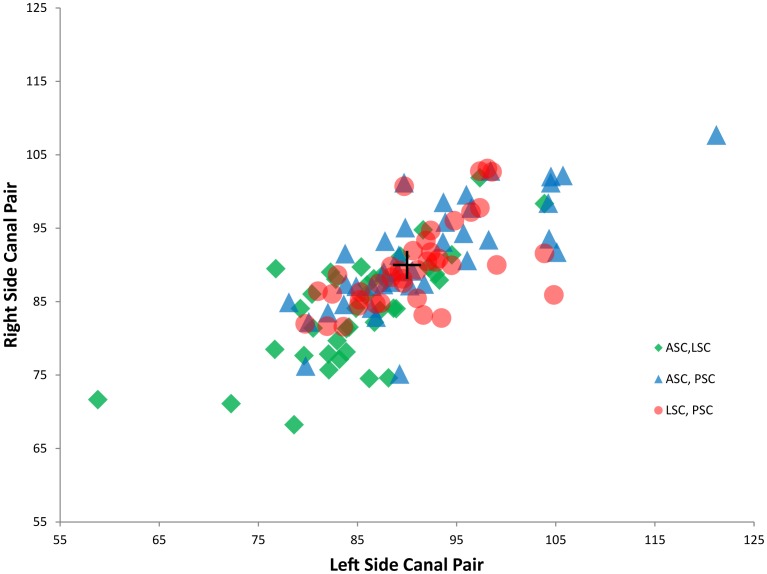
Comparison of ipsilateral canal pair angles from left and right inner ears. Data from Table 2.

We find that the angle between two ipsilateral canals ranges between 58.8° and 121.2° for the species in our sample (Table 2; Figure 3). The smallest canal pair angle in our sample (LASC∡LLSC in *Chrysochloris*) is 31.2° less than 90°, while the largest canal pair angle in our sample (LASC∡LPSC in *Notoryctes*) is 31.2° greater than 90°. The mean angle for all ipsilateral canal pairs across all taxa is 88.9° (st. dev. = 7.8°) and the average deviation from orthogonality for all ipsilateral canal pairs is 6.0° (st. dev. = 5.1°). Similarly, 90_var_ ranges from a low value of 2.3° in *Potorous* to a high value of 15.5° in *Notoryctes* (mean = 6.0°, st. dev. = 2.6°). Of the ipsilateral canal pairs, ASC∡LSC is smallest, with a mean angle of 84.5° (st. dev. = 7.3°) and a 95% mean confidence interval of 83°–86° (i.e., excluding orthogonality). By comparison, mean ASC∡PSC is 91.9° (st. dev. = 8.1°) and mean LSC∡PSC is 90.3° (st. dev. = 5.9°) (Table 3).

In addition to these deviations from orthogonality, our data demonstrate that ipsilateral canal pair angles differ by an average of 4.3° (st. dev. = 2.6°) between the right and left sides of individual specimens. Mean Angle Symmetry_dev_ values range from a low of 0.8° in *Lepus* to a high of 11.6° in *Notoryctes*. Angles between synergistic canal pairs range from 0.5° (*Enhydra* LPSC ∡RASC) to 27.7° (*Caluromys* LLSC ∡RLSC). The mean deviation from coplanarity is 9.5° (st. dev. = 5.2°) for the two ASC∡PSC pairs and 11.2° (st. dev. = 7.5°) for the LSC∡LSC pair (Table 3). The mean deviation of all three synergistic canal pair angles from coplanarity is 10.1° (st. dev. = 6.1°).

### Canal orientation and vestibular sensitivity

**Figure 4:**
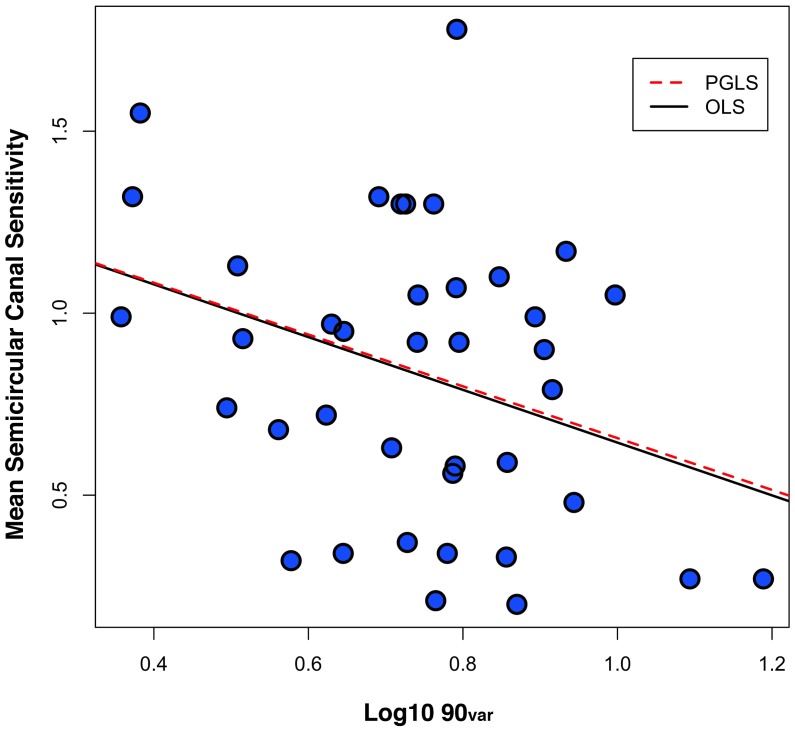
Regressions of mean estimated semicircular canal sensitivity (Sensitivity_ave_) on Log_10_ 90_var_.

As expected, there is a negative correlation between the two estimates of vestibular sensitivity and the three measures of semicircular canal orientation (Table 5). This relationship is significant at P<0.05 for all comparisons except that of Angle Symmetry_dev_ and Sensitivity_max_, which are negatively correlated at P = 0.054. These data demonstrate that species with lower estimated sensitivity to angular accelerations tend to have semicircular canals that deviate more from orthogonality, angle symmetry, and coplanarity. However, the strength of these negative correlations is relatively modest, with correlation coefficients ranging between −0.262 and −0.335 (Table 5). Furthermore, when these relationships are analyzed using PGLS regression to control for phylogenetic non-independence, the results for Angle Symmetry_dev_ and Coplanarity_dev_ are non-significant. By contrast, the PGLS regression of 90_var_ and Sensitivity_ave_ remains significant at P = 0.029 (Figure 4) and the PGLS regression of 90_var_ and Sensitivity_max_ is near significance at P = 0.060. Lambda values for all six comparisons demonstrate that the relationships between these variables do not follow a strict Brownian motion model of evolution, nor are they completely free of the influence of phylogeny (Table 5). According to these results, phylogenetic proximity has the smallest influence on the relationship between Angle Symmetry_dev_ and vestibular sensitivity (λ∼0.64–0.68) and the greatest influence on the relationship between Coplanarity_dev_ and vestibular sensitivity (λ∼0.76–0.79).

## Discussion

Our results reveal that the canonical model of semicircular canal orientation is not strictly correct for a wide range of mammalian species. In our comparative sample, angles between pairs of ipsilateral semicircular canals deviate from orthogonality by an average of 6.0°, corresponding left-right canal pair angles differ by an average of 4.3°, and synergistic canals deviate from coplanarity by an average of 10.1°. Although the angle between any two ipsilateral semicircular canals does approach 90° when data are averaged for all canal pairs across all taxa, it is nevertheless clear that all mammals deviate from canal orthogonality to some degree. For example, *Potorous*, *Pedetes*, and *Wallabia* all have ipsilateral canal pair angles that diverge from 90° by an average of between only 2° and 3°. By contrast, *Notoryctes*, *Chrysochloris*, and *Allactaga* all have ipsilateral canal pair angles that diverge from 90° by an average of nearly 10° or more. These data further underscore the fact that none of the taxa considered in this analysis has truly orthogonal semicircular canals, and that substantial deviations from orthogonality, symmetry, and coplanarity appear to be a common feature of vestibular anatomy in mammals. However, it is also important to acknowledge that our comparative sample does not address questions related to intraspecific variation in canal orientation, so it is currently unclear how representative the values reported here are for each species in our dataset (Tables 1, 2). Our results also demonstrate that the mean angle between the anterior and lateral canals is considerably less than 90° and is 6.4°–7.4° lower than the mean angle between the other two ipsilateral canal pairs. In a functional context, is not presently clear why the angle between ipsilateral anterior and lateral canals is systematically lower than that for other ipsilateral canal pairs, but this finding further reinforces the inaccuracy of general characterizations of semicircular canals as orthogonal [3]–[16].

Previously published studies of semicircular canal morphology in 43 living and fossil species support our general finding that substantial deviations from the canonical model are the norm for mammals (Table 6). When unilateral measurements are considered, these analyses document a range of ipsilateral canal pair angles between 52.2° and 117.0° and an average 90_var_ for all ipsilateral canal pairs of 7.9° (st. dev. = 4.4°). As in the species we examined, the published data also show that across taxa the angle between the anterior and lateral canals (ASC∡LSC mean = 82.6°, st. dev. = 11.3°) is considerably smaller than the angle between the other two ipsilateral canal pairs (ASC∡PSC mean = 93.6°, st. dev. 9.7°; LSC∡PSC mean = 91.4°, st. dev. = 7.4°). The published taxa also show a mean deviation from coplanarity of 16.9° (st. dev. = 6.7°) for both ASC∡PSC pairs and 13.3° (st. dev. = 6.9°) for the LSC∡LSC pair. The mean deviation of all three synergistic canal pair angles from coplanarity is 15.8° (st. dev. = 6.9°).

**Table 6:** Previous semicircular canal pair angle research results.

Taxon	LASC ∡LLSC IPS	LASC ∡LPSC IPS	LLSC ∡LPSC IPS	RASC ∡RLSC IPS	RASC ∡RPSC IPS	RLSC ∡RPSC IPS	LASC ∡RASC CON	LPSC ∡RPSC CON	LASC ∡RPSC SYN	RASC ∡LPSC SYN	LLSC ∡RLSC SYN	n	Sources and Notes
*Atelerix albiventris*	**82.20**	**91.70**	**92.10**									1	Ekdale (2009) [3]c
*Balaenopteridae†*	**71.60**	**105.00**	**75.60**									1	Ekdale (2009) [3]c
*Bathygenys reevesi†*	**86.00**	**99.60**	**91.30**									1	Ekdale (2009) [3]c
*Canis familiaris*	**80.40**	**101.00**	**89.10**									1	Ekdale (2009) [3]c
*Cavia porcellus*	**57.85**	**76.71**	**82.36**	**57.85**	**76.71**	**82.36**			**32.17**	**36.16**	**30.82**	10	Curthoys et al (1975)* [1] a
	*±6.13*	*±5.49*	*±4.74*	*±6.13*	*±5.49*	*±4.74*			*±4.42*	*±4.86*	*±10.05*		
	10.6	7.16	5.76	10.60	7.16	5.76			13.74	13.44	32.61		
*Cavia porcellus*				**91.22**	**91.20**	**85.88**						8	Cox and
				*±6.97*	*±5.78*	*±5.3*							Jeffery
				7.64	6.34	6.17							(2008) [2]a
*Cavia porcellus*	**77.20**	**105.00**	**85.50**									1	Ekdale (2009) [3]c
*Chinchilla*	**76.30**	**90.90**	**103.00**	**78.70**	**91.00**	**101.00**			**15.60**	**14.80**	**14.60**	3	Hullar and Williams (2006)*a
*lanigera*	±2.7	±1.7	±5.7	±5.7	±2.3	±6.9			±1.3	±0.3	±12		
	3.54	1.87	5.53	7.24	2.53	6.83			8.33	2.03	82.19		
*Chrysochloris sp.*	**65.60**	**86.90**	**96.70**									1	Ekdale (2009) [3]c
*Cynocephalus volans*	**92.20**	**90.00**	**91.80**									1	Ekdale (2009) [3]c
*Dasypus novemcinctus*	**62.40**	**67.70**	**87.30**									1	Ekdale (2009) [3]c
*Didelphis virginiana*	**109.00**	**102.00**	**104.00**									1	Ekdale (2009) [3]c
*Elephas†*	**66.30**	**73.70**	**96.70**									1	Ekdale (2009) [3]c
*Equus caballus*	**84.70**	**93.30**	**90.10**									1	Ekdale (2009) [3]c
*Eumetopias jubatus*	**79.70**	**105.00**	**90.60**									1	Ekdale (2009) [3]c
*Felis catus*	**95.60**	**87.90**	**94.10**	**95.60**	**87.90**	**94.10**	**81.10**	**103.80**	**13.90**	**13.90**	**14.40**	3	Ezure & Graf (1984) [1]a
*Felis catus*	**89.62**	**90.21**	**94.23**	**89.62**	**90.21**	**94.23**			**13.92**	**14.49**	**12.49**	7	Blanks et al.
	*±8.71*	*±4.05*	*±3.84*	*±8.71*	*±4.05*	*±3.84*			*±3.99*	*±4.52*	*±9.21*		(1972) [1]a
	9.72	4.49	4.08	9.72	4.49	4.08			28.66	31.19	73.74		
*Felis catus*				**78.36**	**103.34**	**89.49**						8	Cox and
				*±10.05*	*±9.7*	*±6.94*							Jeffery
				12.83	9.39	7.76							(2008) [2]a
*Felis catus*	**76.80**	**91.40**	**96.70**									1	Ekdale (2009) [3]c
*Hemicentetes semispinosum*	**85.40**	**117.00**	**92.60**									1	Ekdale (2009) [3]c
*Homo sapiens*	**90.60**	**94.40**	**90.40**	**90.60**	**94.40**	**90.40**	**103.40**	**83.20**	**15.30**	**15.30**	**11.30**	10	Della
	*±6.2*	*±1.85*	*±4.9*	*±6.2*	*±4*	*±4.9*	*±9.5*	*±9.7*	*±7.2*	*±7.2*	*±6.9*		Santina et
	6.84	2.02	5.42	6.84	4.24	5.42	9.19	11.66	47.06	47.06	61.06		al. (2005)
													[1]c
*Homo sapiens*	**68.24**	**86.16**	**95.75**	**68.24**	**86.16**	**95.75**			**24.56**	**23.73**	**19.82**	10	Blanks et al.
	*±7.55*	*±4.72*	*±4.66*	*±7.55*	*±4.72*	*±4.66*			*±7.19*	*±6.71*	*±14.93*		(1975)* [1]a
	11.06	5.48	4.87	11.06	5.48	4.87			29.28	28.28	75.33		
*Homo sapiens*	**90.50**	**91.70**	**94.52**	**90.50**	**91.70**	**94.52**						7	Hashimoto
	*±2.98*	*±1.85*	*±3.32*	*±2.98*	*±1.85*	*±3.32*							et al. 2005a
	3.29	2.02	3.51	3.29	2.02	3.51							
*Homo sapiens*				**85.30**	**97.14**	**88.96**						6	Cox and
				*±5.81*	*±4.82*	*±6.33*							Jeffery
				6.81	4.96	7.12							(2008) [2] a
*Homo sapiens*	**98.90**	**100.00**	**89.80**									1	Ekdale (2009) [3]c
*Kulbeckia kulbecke* ^†^	**79.90**	**79.90**	**89.60**									4	Ekdale (2009) [1]
*Lepus californicus*	**84.20**	**94.00**	**88.60**									1	Ekdale (2009) [3]c
*Macaca mulatta*	**98.73**	**86.48**	**88.49**	**98.73**	**86.48**	**88.49**			**10.67**	**11.18**	**2.24**	10	Blanks et al.
	*±5.39*	*±3.43*	*±3.91*	*±5.39*	*±3.43*	*±3.91*			*±3.55*	*±3.12*	*±0.77*		(1985) [1] a
	5.46	3.97	4.42	5.46	3.97	4.42			33.27	27.91	34.38		
*Macaca mulatta*	**83.10**	**100.00**	**89.00**									1	Ekdale (2009) [3]c
*Macroscelides proboscideus*	**100.00**	**90.70**	**73.50**									1	Ekdale (2009) [3]c
*Manis tricuspis*	**77.00**	**84.80**	**88.60**									1	Ekdale (2009) [3]c
*Mus C57BL/6J*	**92.56**	**99.02**	**101.17**	**91.55**	**99.26**	**101.26**			**17.61**	**17.66**	**9.14**	4	Calabrese
	*±1.93*	*±1.46*	*±0.97*	*±1.02*	*±1.29*	*±0.96*			*±2.73*	*±1.43*	*±0.98*		and Hullar
	2.09	1.47	0.96	1.11	1.30	0.95			15.50	8.10	10.72		(2006)a
*Mus CBA/CaJ*	**96.82**	**89.65**	**102.29**	**95.47**	**88.94**	**102.05**			**11.00**	**14.79**	**10.42**	4	Calabrese
	*±5.73*	*±2.51*	*±1.86*	*±1.94*	*±1.98*	*±2.14*			*±1.24*	*±2.11*	*±3.8*		and Hullar
	5.92	2.80	1.82	2.03	2.23	2.10			11.27	14.27	36.47		(2006)a
*Mus musculus*				**76.63**	**101.54**	**96.08**						9	Cox and
				*±6.02*	*±6.32*	*±6.06*							Jeffery
				7.86	6.22	6.31							(2008) [2]a
*Mus musculus*	**88.80**	**94.40**	**95.60**									1	Ekdale (2009) [3]c
*Nycteris grandis*	**85.90**	**112.00**	**94.90**									1	Ekdale (2009) [3]c
*Nycticebus coucang*		**88.60**			**88.60**		**68.20**	**114.40**	**23.10**	**23.10**		3	Matano et al. (1985) [1]
*Orycteropus afer*	**78.50**	**91.90**	**95.70**									1	Ekdale (2009) [3]c
*Oryctolagus cuniculus*	**79.80**	**76.60**	**75.50**	**79.80**	**76.60**	**75.50**	**90.50**	**116.50**	**13.60**	**13.60**	**8.60**	3	Ezure & Graf (1984) [1]a
*Oryctolagus*	**79.36**	**71.36**	**75.85**	**79.36**	**71.36**	**75.85**	**85.76**	**47.54**	**26.78**	**26.78**	**15.32**	7	Mazza and
*cuniculus*	*±9.4*	*±4.4*	*±6.7*	*±9.4*	*±4.4*	*±6.7*	*±5.6*	*±5.3*	*±6.8*	*±6.8*	*±7.2*		Winterson
	11.84	6.17	8.83	11.84	6.17	8.83	6.53	11.15	25.39	25.39	47.00		(1984)* [1]a
*Oryctolagus*				**81.73**	**97.05**	**97.52**						9	Cox and
*cuniculus*				*±11*	*±5.6*	*±9.82*							Jeffery
				13.46	5.77	10.07							(2008) [2]a
*Procavia capensis*	**87.40**	**112.00**	**87.40**									1	Ekdale (2009) [3]c
*Pteropus lyelli*	**84.90**	**98.30**	**90.40**									1	Ekdale (2009) [3]c
*Rattus norvegicus*				**73.35**	**97.57**	**98.12**						8	Cox and
				6.37	4.80	10.89							Jeffery
				8.68	4.92	11.10							(2008) [2]a
*Rattus norvegicus*	**97.60**	**94.20**	**93.50**	**97.60**	**94.20**	**93.50**			**9.90**	**9.90**	**8.00**	14	Blanks and Torigoe (1989) [1]b
*Rhinolophus ferrumequinum*	**79.90**	**104.00**	**87.90**									1	Ekdale (2009) [3]c
*Saimiri sciureus*	**90.43**	**87.02**	**89.95**	**90.43**	**87.02**	**89.95**			**12.53**	**14.80**	**15.45**	10	Blanks et al.
	*±6.94*	*±4.22*	*±5.08*	*±6.94*	*±4.22*	*±5.08*			*±5.55*	*±5.37*	*±5.98*		(1985) [1]a
	7.67	4.85	5.65	7.67	4.85	5.65			44.29	36.28	38.71		
*Sciurus*				**78.97**	**89.52**	**104.41**						5	Cox and
*carolinensis*				*±6.61*	*±4.4*	*±9.64*							Jeffery
				8.37	4.92	9.23							(2008) [2]a
*Sorex monticolus*	**75.30**	**89.60**	**89.30**									1	Ekdale (2009) [3]c
*Sus scrofa*	**82.80**	**96.00**	**87.90**									1	Ekdale (2009) [3]c
*Sylvilagus floridanus*	**92.70**	**97.50**	**77.90**									1	Ekdale (2009) [3]c
*Tadarida brasiliensis*	**74.70**	**98.40**	**98.40**									1	Ekdale (2009) [3]c
*Tarsius bancanus*		**91.80**			**91.80**		**73.80**	**102.80**	**14.50**	**14.50**		3	Matano et al. (1985) [1]
*Trichechus manatus*	**52.20**	**84.90**	**86.30**									1	Ekdale (2009) [3]c
*Tupaia glis*	**82.30**	**106.00**	**102.00**									1	Ekdale (2009) [3]c
*Tursiops truncatus*	**52.20**	**84.90**	**77.50**									1	Ekdale (2009) [3]c
*Ukhaatherium gobiensis* ^†^	**88.80**	**105.00**	**88.40**									1	Ekdale (2009) [3]c
*Zalambdalestes lechei^†^*	**81.00**	**93.60**	**85.60**									4	Ekdale (2009) [3]c
Zhelestid^†^	**88.80**	**96.80**	**93.10**									7	Ekdale (2009) [3]c
**Mean**	**82.46**	**93.21**	**90.37**	**84.27**	**90.42**	**92.35**	**83.79**	**94.71**	**17.01**	**17.65**	**13.28**		
*Standard Dev.*	*±11.87*	*±10.14*	*±7.19*	*±10.41*	*±7.82*	*±7.85*	*±12.52*	*±25.95*	*±6.59*	*±6.94*	*±6.85*		
Coeff. Variation	14.39	10.88	7.96	12.35	8.64	8.50	14.94	27.40	38.77	39.35	51.63		
95% Confidence	80.78	91.80	89.35	82.00	88.79	90.64	78.68	84.11	15.31	15.85	11.38		
Interval of Mean	84.13	94.61	91.39	86.54	92.05	94.07	88.90	105.30	18.71	19.44	15.88		

Where sides were averaged for results, those results were recorded in both the right and left sides. Each entry includes standard deviation and coefficient of variation (in italics) if available. Total specimens measured, 199; 44 different species reported. Abbreviations for notes: [**1**] sides averaged, [**2**] MRI study based on one side only, [**3**] mostly left labyrinths used, but specimens with right sides indeterminate,

a least squares fit and eigenvector analysis, b null point technique - mounting and orientation that gives no afferent response from a particular canal; c visual fit,

**CON** contralateral canals, **IPS** ipsilateral canals, **n** number of specimens examined, **SYN** synergistic canals,

* **ASC∡LSC** corrected for internal angles.

From a practical standpoint, these data have important implications for the use of lateral canal orientation as an indicator of the horizontal plane in reconstructions of head posture in fossil mammals [31], [39], [51]–[53]. In our comparative sample, 7 of the 39 species have right and left lateral canals that deviate from coplanarity by more than 20° (Table 2). This large amount of bilateral variation in lateral canal orientation within individual specimens suggests that the lateral canal is an imprecise indicator of habitual head orientation (resting or active) in fossil species, particularly if reconstructions are based on unilateral measurements of semicircular canals [54].

Our data also generally confirm the expectation that there are important functional consequences of the degree to which a species' vestibular anatomy deviates from the canonical model. In particular, deviations from canal orthogonality (as measured by 90_var_) are negatively correlated with both of our estimates of vestibular sensitivity (Table 5). This result is most pronounced for the relationship between 90_var_ and mean sensitivity (Sensitivity_ave_, Figure 4). As noted previously, the values for Sensitivity_ave_ reported here are determined entirely by canal radii of curvature (Figure 2), so the significant negative relationship between 90_var_ and Sensitivity_ave_ is unrelated to our methods for estimating canal sensitivity. By the same token, deviations from orthogonality tend to increase the maximum vestibular sensitivity (i.e., result in higher Senstivity_max_ ∶ OSensitivity_max_ ratio; Figure 2) according to the methods employed here. In other words, based on our estimates of canal sensitivity, constraining canals to be perfectly orthogonal always decreases Senstivity_max_ (Figure 2, Table 4). Accordingly, our results for 90_var_ and Sensitivity_max_ (Table 5) should be interpreted with caution because estimated maximum vestibular sensitivity is determined both by the radii and orientations of canals. Nevertheless, our analysis demonstrates that as the average deviation from canal orthogonality in our interspecific comparative sample increases (i.e., higher 90_var_), the mean estimated sensitivity to angular head accelerations tends to decrease. As a result, species with more orthogonal semicircular canals tend to have higher mean vestibular sensitivity than species with less orthogonal semicircular canals (Figure 4). Although canal radius of curvature remains a major determinant of semicircular canal sensitivity, these findings imply that selection for greater sensitivity to angular head accelerations may influence semicircular canal orthogonality.

We also find that deviations from canal angle symmetry and coplanarity are negatively correlated with estimated vestibular sensitivity. However, these correlations are weaker and more strongly influenced by phylogeny compared to the results for orthogonality. As a result, phylogenetically controlled analyses of the relationship between estimated vestibular sensitivity and both angle symmetry and coplanarity are not significant (Table 5). These results do not necessarily imply the absence of a functional relationship between canal angle symmetry or coplanarity and vestibular sensitivity, but they do indicate that there is a strong phylogenetically correlated influence on these relationships.

In this context, it is also noteworthy that the obligate fossorial genera in our analysis (*Notoryctes*, *Chrysochloris*, *Talpa*, and *Heterocephalus*) show greater deviations from canal orthogonality than most non-fossorial genera (Table 2). Indeed, the average 90_var_ value for the 4 fossorial taxa in our sample (mean = 11.0°; st. dev. = 3.2°) is twice that of non-fossorial taxa (mean = 5.5°; st. dev. = 1.8°). *Notoryctes*, *Chrysochloris*, *Talpa*, and *Heterocephalus* also share comparatively low estimates of semicircular canal sensitivity (fossorial taxa: Sensitivity_ave_ = 0.27–0.48; Sensitivity_max_ = 0.38–0.59; non-fossorial taxa: Sensitivity_ave_ mean = 0.89, st. dev. = 0.38; Sensitivity_max_ mean = 1.10, st. dev. = 0.47; Table 4). While this sample of fossorial genera is small, it is also taxonomically diverse, including a marsupial (*Notoryctes*), an afrothere (*Chrysochloris*), a eulipotyphlan (*Talpa*), and a rodent (*Heterocephalus*). These data therefore suggest that low degrees of semicircular canal orthogonality and relatively low sensitivity to angular accelerations may have evolved concurrently with a fossorial lifestyle at least 4 times in mammals. Nonetheless, it is not functionally clear why lower degrees of canal orthogonality would be associated with a burrowing lifestyle.

Our findings are consistent with those of Billet et al. [55], who report highly variable and non-orthogonal ipsilateral canal pair angles in three-toed sloths (*Bradypus variegatus*). Billet et al. suggested that such high variability is the result of diminished selection pressure in slower-moving mammals to maintain orthogonal semicircular canals. Furthermore, the negative relationship between angular head velocities and 90_var_ observed by Malinzak et al. [33] accords well with our finding of a negative relationship between mean vestibular sensitivity and 90_var_ (Figure 4). The combined results of both studies thus show that species with the greatest deviations from canal orthogonality tend to experience slower head rotations during locomotion [33] and to have less sensitive semicircular canals (Figure 4). Although Malinzak et al. [33] based their conclusions on a smaller sample of 11 primate species, their analysis is the only comparative study to date that directly measured angular head velocities produced during locomotion. These authors further concluded that species which regularly encounter higher angular head accelerations during locomotion require more orthogonal canals in order to have more uniform sensitivity to angular accelerations in three dimensions. Here we have shown that the degree to which semicircular canals approach orthogonality is correlated with mean estimated sensitivity to angular accelerations, and that mean sensitivity in turn is solely determined by canal radius of curvature. These findings reinforce the conclusion that both the radii and orientations of the semicircular canals may be influenced by selection related to forces generated during locomotion.
